# The kinetics of cellular and humoral immune responses of common carp to presporogonic development of the myxozoan *Sphaerospora molnari*

**DOI:** 10.1186/s13071-019-3462-3

**Published:** 2019-05-06

**Authors:** Tomáš Korytář, Geert F. Wiegertjes, Eliška Zusková, Anna Tomanová, Martina Lisnerová, Sneha Patra, Viktor Sieranski, Radek Šíma, Ana Born-Torrijos, Annelieke S. Wentzel, Sandra Blasco-Monleon, Carlos Yanes-Roca, Tomáš Policar, Astrid S. Holzer

**Affiliations:** 1 Institute of Parasitology, Biology, Centre of the Czech Academy of Sciences, České Budějovice, Czech Republic; 2 Faculty of Fisheries and Protection of Waters, South Bohemian Research Center of Aquaculture and Biodiversity of Hydrocenoses, University of South Bohemia, České Budějovice, Czech Republic; 3 Aquaculture and Fisheries Group, Wageningen Institute of Animal Sciences, Wageningen University & Research, Wageningen, The Netherlands; 4 Faculty of Science, University of South Bohemia in České Budějovice, České Budějovice, Czech Republic; 5 Faculty of Engineering and Natural Sciences, Johannes Kepler University, Linz, Austria; 6 Cell Biology and Immunology Group, Wageningen Institute of Animal Sciences, Wageningen University & Research, Wageningen, The Netherlands

**Keywords:** Host–parasite interaction, Myxozoa, *Sphaerospora molnari*, Teleost, *Cyprinus carpio*, B cells, IgM, Cytokines

## Abstract

**Background:**

*Sphaerospora molnari* is a myxozoan parasite causing skin and gill sphaerosporosis in common carp (*Cyprinus carpio*) in central Europe. For most myxozoans, little is known about the early development and the expansion of the infection in the fish host, prior to spore formation. A major reason for this lack of information is the absence of laboratory model organisms, whose life-cycle stages are available throughout the year.

**Results:**

We have established a laboratory infection model for early proliferative stages of myxozoans, based on separation and intraperitoneal injection of motile and dividing *S. molnari* stages isolated from the blood of carp. In the present study we characterize the kinetics of the presporogonic development of *S. molnari*, while analyzing cellular host responses, cytokine and systemic immunoglobulin expression, over a 63-day period. Our study shows activation of innate immune responses followed by B cell-mediated immune responses. We observed rapid parasite efflux from the peritoneal cavity (< 40 hours), an initial covert infection period with a moderate proinflammatory response for about 1–2 weeks, followed by a period of parasite multiplication in the blood which peaked at 28 days post-infection (dpi) and was associated with a massive lymphocyte response. Our data further revealed a switch to a massive anti-inflammatory response (up to 1456-fold expression of *il-10*), a strong increase in the expression of IgM transcripts and increased number of IgM^+^ B lymphocytes, which produce specific antibodies for the elimination of most of the parasites from the fish at 35 dpi. However, despite the presence of these antibodies, *S. molnari* invades the liver 42 dpi, where an increase in parasite cell number and indistinguishable outer cell membranes are indicative of effective exploitation and disguise mechanisms. From 49 dpi onwards, the acute infection changes to a chronic one, with low parasite numbers remaining in the fish.

**Conclusions:**

To our knowledge, this is the first time myxozoan early development and immune modulation mechanisms have been analyzed along with innate and adaptive immune responses of its fish host, in a controlled laboratory system. Our study adds important information on host–parasite interaction and co-evolutionary adaptation of early metazoans (Cnidaria) with basic vertebrate (fish) immune systems and the evolution of host adaptation and parasite immune evasion strategies.

**Electronic supplementary material:**

The online version of this article (10.1186/s13071-019-3462-3) contains supplementary material, which is available to authorized users.

## Background

Myxozoans are possibly the oldest metazoan parasites to diverge early in the evolution of free-living cnidarians (650 Mya), initially as parasites of annelid and bryozoans [1]. The acquisition of fish as secondary hosts later in evolution facilitated alternative transmission and dispersion strategies resulting in a distinct success. The conquest of a more evolved intermediate host, however, imposed important challenges on myxozoans, especially because of the hosts’ ability to induce parasite-specific adaptive immune responses [2, 3]. While the innate immunity of annelids has a limited capacity to “remember” pathogens, fish are vertebrates and possess a sophisticated system of antigen-specific B and T cells capable of developing memory with the aim to respond more vigorously and effectively to repeated exposures [4]. Since the initial encounter of myxozoans with fish approximately 440 Mya, these two groups co-diversified and co-evolved by continuous adaptation of immune responses and evasion strategies. This possibly contributed to the reduced antagonism of the parasite [5], explaining why out of the approximately 2500 described myxozoan species, only a small percentage are represented by serious pathogens [6]. This small percentage, however, causes severe diseases and important economic losses in aquaculture and wild fish stocks. Notably, the highest severity of the diseases is observed in newly acquired species, which do not have a common history with the parasite. With this regard, it is noteworthy that the proliferation and disease severity of myxozoans has been linked to increasing water temperature and epidemical models predict spread of disease and major outbreaks as a result of climate change [7, 8]. Resulting (re-)emergence and dissemination of myxozoan diseases into new areas therefore represents a major threat. Although concentrated efforts have resulted in significant advances in our understanding of the biology, transmission and pathogenicity of these parasites, detailed knowledge about host–parasite interaction throughout disease progression, and the ability of the host’s immune system to eliminate the parasite and protect from re-infection is still limited [9].

A major reason for limited information on host–parasite interactions is the absence of available *in vivo* models. Only 55 life-cycles have been elucidated to date [1] and very few (3–4) are continuously perpetuated in research laboratories, as their maintenance is laborious and time-consuming [10]. We have recently established the first continuous *in vivo* model system for myxozoan pre-sporogonic proliferative stages. The model employs *Sphaerospora molnari*, a common parasite of carp in pond cultures and natural freshwater habitats in central and eastern Europe [11, 12]. In natural infections, *S. molnari* invades the epithelia of skin and gills where it forms spores intracellularly [13]. Importantly, prior to spore formation, *S. molnari* and other species from the same phylogenetic clade multiply in fish blood [14, 15]. The pluricellular blood stages, initially termed unidentified blood objects (UBOs) [16] proliferate rapidly and show a unique motility which may help them to evade contact with host immune cells [17]. Our research model takes advantage of these blood stages and bypasses problems related to the development and spore maturation in the invertebrate host, by transferring the parasite *via* intraperitoneal injection from fish to fish, in a similar fashion as performed by Ibarra et al. [18, 19] for the myxozoan *Ceratonova shasta*, using parasite stages obtained from ascites of infected fish. This allows for functional analyses of early parasite establishment, migration, development, multiplication and a better understanding of the cross-talk between host and pathogen, under controlled laboratory conditions.

Previously, a number of studies have aimed to elucidate various aspects of the teleost immune response towards myxozoans. Although direct comparison is complicated, they have provided valuable information on the activation of both innate and adaptive immune responses [9, 20, 21]. As with other pathogens, myxozoans avoid the immune system by intracellular proliferation or by exploiting immune privileged sites, e.g. the nervous tissues, where the presence of leukocytes and immune mediators is limited [22–25]. Despite these efforts, infections lead to the recruitment of macrophages, neutrophils and other granulocytes to infected tissues [26–28] and increased production of innate humoral factors including peroxidases, lysozyme or complement, which are involved in killing these parasites [9]. The changes in gene expression induced by myxozoan infections are often only weak or transient with downregulation of pro-inflammatory cytokines and a dominant anti-inflammatory phenotype [29–32]. With regard to adaptive immunity, a number of reports demonstrated an increase in the number of B cells in infected tissues and presence of myxozoan-specific antibodies in the sera or mucus of survivor fish (summarized in [9, 21, 33]). Although the development of these antibody responses is slower than to other antigens, some antibodies showed partial to complete protection to re-infection [34, 35]. Most of the available data, however, focuses on myxozoans within tissues such as intestine or kidney, and the information on developmental stages in the blood is scarce. Undoubtedly, living in the blood stream, exposed to the full array of humoral and cellular immune effector mechanism, must represent a considerable challenge for the parasite survival, and while motility may explain its escape from effector leukocytes [17], it is certainly not the only evasion strategy *S. molnari* exploits. To date, information on parasite replication and changes in the innate and adaptive immunity of the host is not known. The key aims of the presented study thus were to broaden our understanding of fish–myxozoan interaction by analyzing simultaneously and over time, the kinetics of *S. molnari* presporogonic infection in the host’s body tissues together with an overall activation of the immune system on a cellular and molecular level, over a 63 day period. To shed some light on these questions, we analyzed the expression of selected pro- and anti-inflammatory cytokines in the head kidney, namely *il-1β*, *il-6*, *tnfα*, *ifnγ*, *il-11* and *il-10*, and investigated changes in the composition of blood leukocytes, as a first indicator of activation of the immune system. Furthermore, to elucidate the development of adaptive immunity, B cell kinetics and the secretion of specific antibodies in the blood were analyzed throughout the experiment.

## Results

### Parasite kinetics over time: Acute vs chronic infection and liver as an unknown reservoir

**Fig. 1:**
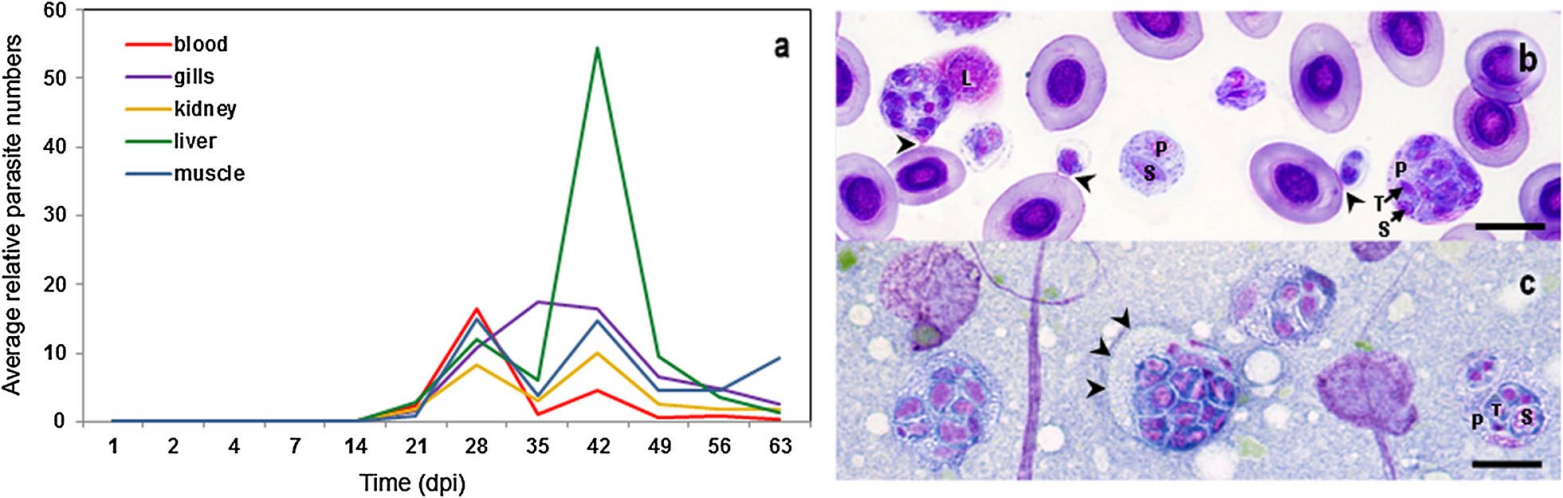
**a** Quantification and localization of *S. molnari* in different organs of common carp, over 63 days post-intraperitoneal-injection (dpi), using qPCR (based on parasite *SSU* rDNA sequence copies); numbers are relative to the sample with the highest value (100; a liver sample from 42 dpi). **b**, **c** Kwik–Diff-stained cell preparations. **b** Blood smear showing multicellular parasite stages in amongst red blood cells and a lymphocyte (L); arrowheads indicate attachment sites to erythrocytes. **c** Liver imprint with larger, multicellular parasite stages, one showing a refractile protruding edge (arrowheads). Cell-in-cell composition is indicated in some parasite stages in **b** and **c**. *Abbreviations*: P, primary cell; S, secondary cell; T, tertiary cell. *Scale-bars*: **b**, **c**, 20 µm

The combination of a controlled infection study using our *S. molnari* laboratory model and a specific qPCR assay which is sensitive enough to consistently detect two parasite stages in a 4 µl blood DNA extraction (data not shown), allowed us to study the number and distribution of *S. molnari* in different organs during 63 days post-intraperitoneal injection (Fig. 1b; Additional file 1: Figure S1), thereby elucidating the kinetics of infection and identifying previously unknown parasite locations. *Sphaerospora molnari* was first detected after a latency period of two to three weeks. At 14 days post-infection (dpi), *S. molnari* was quantifiable by qPCR in one out of five experimentally infected fish. At 21 dpi, all fish tested *S. molnari*-positive by qPCR and by microscopy (parasites detected in the leukocyte fraction of blood spun in hematocrit capillaries). The highest peak of parasite numbers in the blood was determined at 28 dpi (Fig. 1a). At this time point, parasite *SSU* rDNA copy numbers in different organs were similar or lower than those in the blood and it can be assumed that *S. molnari* is represented mainly by proliferative blood stages in the vascular system of these organs. After a decline in all organs but the gills, 35 dpi, parasite numbers showed a second peak of abundance in the blood, 42 dpi, coincident with higher parasite numbers in kidney, muscle, gills and the liver. Notably, in the liver, the maximum *S. molnari* concentrations were determined, approx. 7 times higher than those detected in the blood on the same day, and 5 times higher than during peak blood proliferation, 28 dpi. The stages in the liver represent a previously unknown parasite location in the host. At 56 and 63 dpi, parasite numbers showed a significant decline (Kruskal-Wallis H-test, *χ*^2^ = 6.731, *df* = 8, *P* < 0.001) and the acute stage infection changes to a chronic type. Despite noticeable *S. molnari* gill invasion, 35–42 dpi, we did not observe spore formation in the gills at this time or at any other time point during the experiment.

In Kwik–Diff-stained blood smears and imprints, the cellular composition of *S. molnari* life-cycle stages was studied. Blood stages (BS) were composed predominantly of small bicellular units (one primary cell harboring one secondary cell); only 17% were larger with 3–20 cells (primary cell containing various secondary cells and sometimes secondary-tertiary cell doublets; Fig. 1b). Until 28 dpi, BS were composed of 1–9 cells; the first fish to show elevated numbers of *SSU* rDNA copies in the liver (relative copy number of 11.9, Fig. 1) had larger BS stages with up to 20 cells, which also occurred on most of the subsequent days (49, 56 and 63 dpi). However, no statistically significant changes in the cellular composition of *S. molnari* BS over time were determined (Kruskal-Wallis H-test, *χ*^2^ = 3.527, *df* = 4, *P* = 0.563), similar to a previous study [36] (referred to as ‘Csaba cells’ but likely representing *S. molnari*). BS were often found in contact with host erythrocytes (Fig. 1b, arrowheads) but never within them. In Kwik–Diff-stained imprints of the liver, liver stages (LS) were found to be considerably larger in size than BS and composed of an average of 7 cells (Fig. 1c), but with a range in cellularity identical to that of blood stages (2–20 cells per stage). In LS, secondary cells often occurred in pairs in the cytoplasm of primary cells. Tertiary cells were present only in stages with 6 or more secondary cells and only single tertiary cells were present inside individual secondary cells, the same as in BS. Some parasites showed a separation in a refractile ecto- and more granular endoplasm, usually related to migratory cell motility *via* a protruding edge (Fig. 1c; [37, 38]). The number of parasite stages detected on Kwik–Diff-stained slides of blood smears correlated significantly with the values obtained by qPCR (linear regression, *r*^2^ = 0.2282, *P* = 0.0182) and allowed the calculation of absolute numbers: BS on 28 dpi reached an average density of 9,390 and a maximum density of 11,350 BS/µl of blood. Based on Ct values and average cellular composition up to 29,480 LS/µg liver tissue were estimated.

BS of *S. molnari* stained with carboxyfluorescein succinimidyl ester (CSFE) were detectable in peritoneal lavages 16 h post-infection (hpi) (3.16 ± 0.58% of cells) to 40 hpi (0.07 ± 0.11 of cells), but not at 7 or 21 dpi. BS were also detected in the spleen at very low levels (0–12 cells/100,000 cells) but not in head kidney or blood, 16 hpi. At 21 dpi, one out of three fish was microscopically positive for *S. molnari* BS in the blood but all parasites were CSFE-negative. This indicates that, at this time point, that the parasites had undergone more than 7 cell divisions resulting in 1/128th of their original fluorescence and limiting their cytometric detection [39].

### Host reaction to infection: macroscopic changes and massive lymphocyte response

**Fig. 2:**
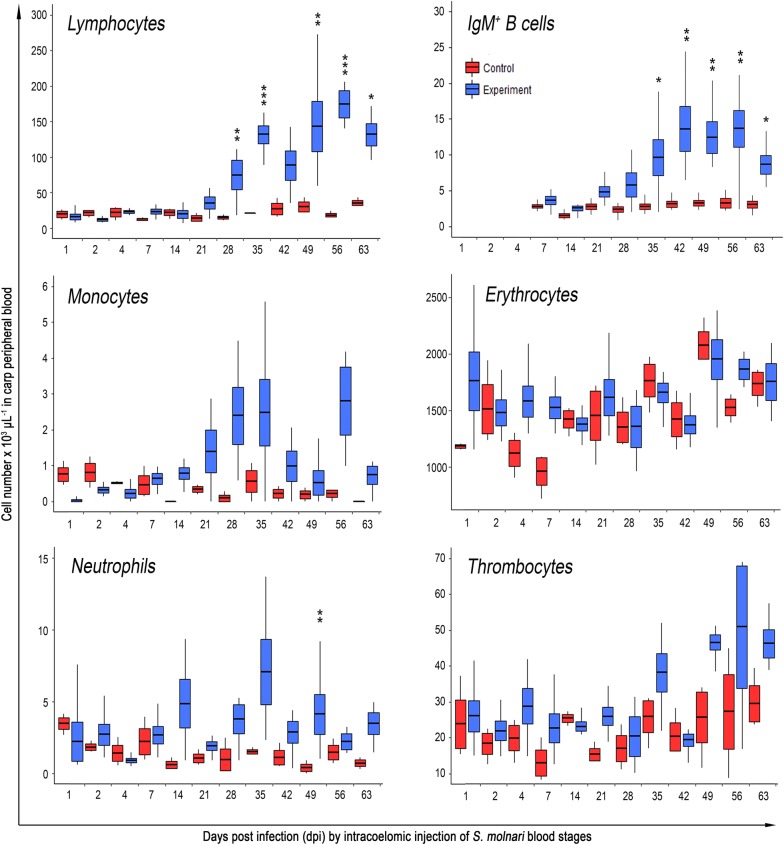
Variation of total erythrocyte, lymphocyte, IgM^+^ B cell, monocyte, neutrophil and thrombocyte numbers as a response of common carp to intraperitoneal *S. molnari* blood stage injection. Data based on Hayem-diluted blood (erythrocytes) and differential blood cell counts (2 × 100 leukocytes + thrombocytes) counted on Kwik–Diff-stained blood smears or by flow cytometry (IgM^+^ B cells; repeat experiment). Note the highly significant increase in lymphocytes from 28 dpi and IgM^+^ B cells from 35 dpi. Statistical analyses are based on data-specific LMM developed in R with significance levels *P < 0.05, **P < 0.01 and ***P < 0.001 relative to the control group. Box plots show mean ± standard deviation (box) with maximum and minimum ranges (lines)

Previously, systemic pathological changes causing massive swelling of hematopoietic organs of common carp were reported for *S. dykovae* infections [11, 40, 41]; however, the presence of other myxozoans in these cases cannot be excluded [42]. In the isolated *S. molnari* infection model, we observed macroscopic changes from 42 dpi onwards and until the end of the experiment, with head kidneys, trunk kidneys and livers being visibly enlarged in all experimental fish. Additionally, and exclusively on 21 dpi, the first day where BS were present in all experimental fish, we noted a blood color change from dark red to brown, without detecting hemolysis in the plasma of centrifuged blood. Furthermore at 21 and 28 dpi, a large percentage of erythrocytes showed cytoplasmic vacuolization and an unusually high number of immature erythrocytes were present. However, erythrocyte number, hematocrit and hemoglobin concentration differed significantly from control fish only at three time points: at 4 and 7 dpi an unexpected decrease of these values was observed in the control group (LM erythrocytes: *F*_(23,68)_ = 3.05, *P* = 0.06, 4 and 7 dpi; LM hematocrit: *F*_(23,65)_ = 4.17, *P* = 0.01, 4 dpi; LM hemoglobin: *F*_(23,68)_ = 3.82, *P* = 0.03, 4 and 7 dpi) and at 56 dpi hematocrit and erythrocyte numbers were meaningfully higher in infected fish than in the control group (Fig. 2, erythrocytes; Additional file 1: Figure S2, hemoglobin and hematocrit).

In the blood, thrombocytes and all leukocyte types showed a substantial increase over time in response to *S. molnari* infection in carp (Fig. 2) with a maximum increase in lymphocytes (up to 13-fold), especially B cells and monocytes (up to 13-fold), followed by neutrophils (up to 10-fold) and thrombocytes (up to 3-fold), in individual fish. Thrombocyte numbers were higher in infected fish on all days except 1, 2, 14 and 42 dpi (Fig. 2). From 35 dpi onwards, we observed an increase in the number of thrombocytes, with the exception of 42 dpi, when *S. molnari* resided in the liver and fewer thrombocytes were detected in the blood. This decrease at 42 dpi and during the occurrence of LS was also noticed in other immune cells such as lymphocytes and monocytes, while less obvious in neutrophils. Neutrophil and monocyte numbers (Fig. 2) increased from 14 dpi onwards and remained elevated until the end of the experiment. Neutrophils first peaked at 14 dpi and then reached their maximum at 35 dpi, when *S. molnari* blood stages are reduced in the blood. Monocytes also first increased 14 dpi and were considerably elevated at 21, 28, 35 and 56 dpi, while showing a decrease towards the end of the experiment (63 dpi). As the most obvious cellular response to *S. molnari* infection, the number of lymphocytes in infected fish increased massively, from 21 dpi, when *S. molnari* appeared for the first time in the blood. Numbers thereafter increased considerably, with a significant difference to the control group at all time points except for 42 dpi, and a slight decrease noticeable at 63 dpi, similar to monocytes and coincident with the initiation of a chronic low-level infection by *S. molnari* at 49 dpi.

### IgM expression and B cell responses indicate specific acquired immunity but point to a polyclonal B cell activation

**Fig. 3:**
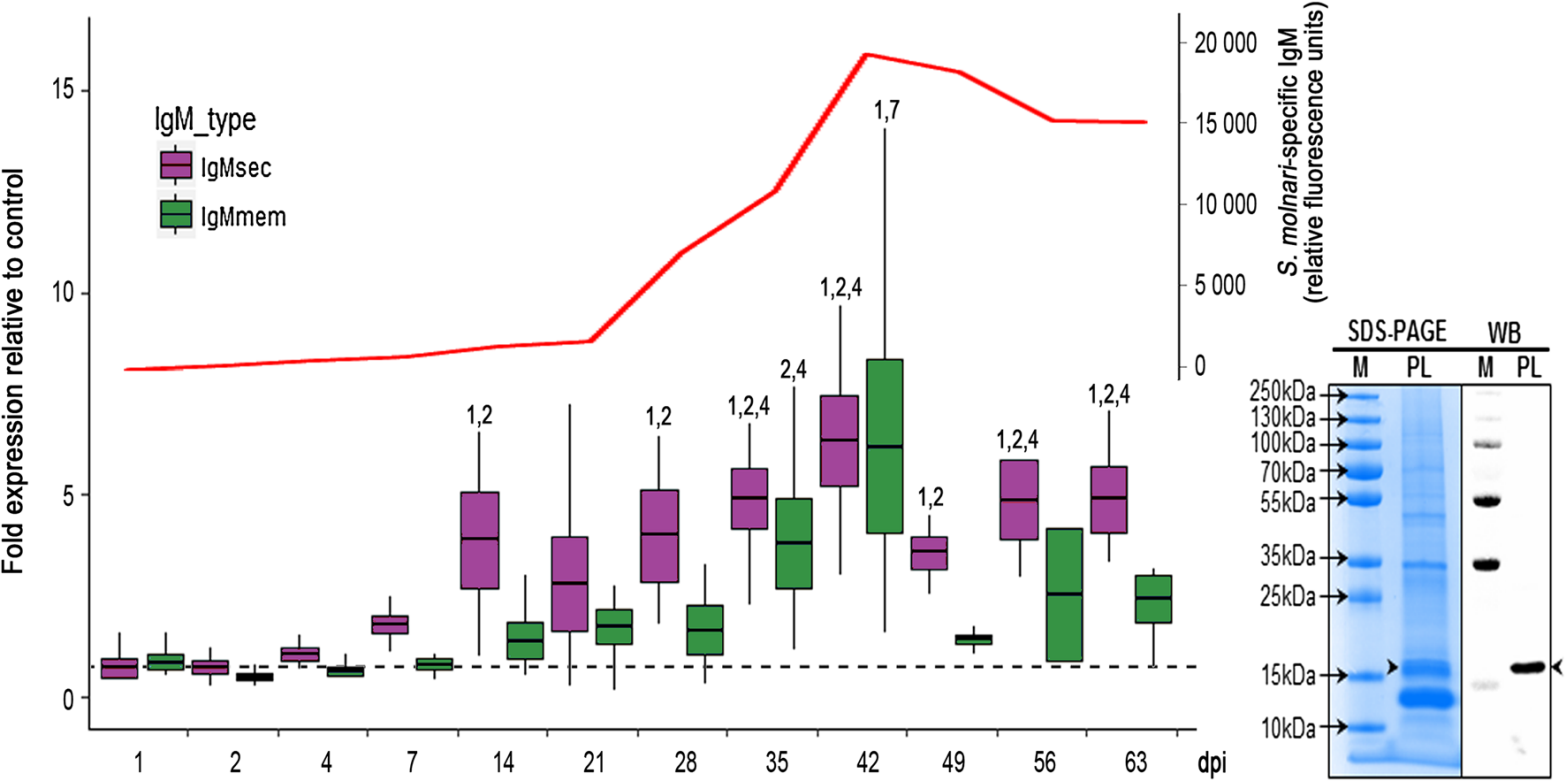
Variation in IgM expression over time as a response to *S. molnari* infection of common carp, estimated by differential TaqMan qPCR assays based on the variable carboxy terminus of the IgM heavy chain of membrane bound monomeric IgM (*IgMmem*) and secretory tetrameric IgM (*IgMsec*), shown in box plots (fold change expression relative to the control group, left x-axis). Statistical analyses are based on data-specific LMM developed in R with significance differences shown relative to other sampling days (numbers above box plot; significance levels detailed in supplemental data 3). Box plots show mean ± standard deviation (box) with maximum and minimum ranges (lines). The red line indicates the presence of *S. molnari*-specific IgM as measured by the intensity of fluorescence (right x-axis) of a 16 kDa parasite band of parasite lysates electrophorized on protein gels, blotted and exposed to sera of infected fish and labelled with anti-carp IgM antibody; right bottom corner: Coomassie-stained gel (blue) and immunoblot (black and white) identifying the 16kDa IgM-binding parasite protein. *Abbreviations*: M, marker; PL, parasite lysate; WB, western blot

We focused on the changes in the expression of systemic IgM, the kinetic of IgM^+^ B cells and the presence of specific IgM in the serum of infected fish. IgM gene expression was analyzed in the head kidney, with differential TaqMan assays for *IgMmem* and *IgMsec*. We demonstrated that *IgMmem* expression (Fig. 3; green box plots) was higher than in the control group, from 21 dpi onwards and increased steadily until 42 dpi (significantly higher when compared from 1 to 7 dpi, LM: *F*_(11,43)_ = 4.9, *P* < 0.01), where it peaked at > 14-fold expression in individual fish and decreased considerably to close to normal levels thereafter. The prominent role of IgM^+^ B cells was further supported by a growing number of the IgM^+^ B cells in peripheral blood. Thus, while the number of B cells remained stable in the blood of fish injected with phosphate-buffered saline (PBS) and ranged between 1.3 and 3 × 10^3^ IgM^+^ lymphocytes/µl of blood, the infection induced an increase from 21 dpi onwards, peaking at 13 × 10^4^ IgM^+^ B cells/µl of blood (approx. 60-fold increase), at 56 dpi (Fig. 2).

We further investigated whether the increased number of B cells was linked to an elevated secretion of IgM into the serum. The results of the qPCR analysis indicated that *IgMsec* expression in the head kidney (Fig. 3; purple box plots) increased significantly from 14 dpi onwards, with an initial peak at 14 dpi (approx. 4-fold expression) and highest levels detected at 42 dpi (up to 10-fold expression in individual fish) (LM: *F*_(11,44)_ = 8.2, *P* < 0.001 to 0.049). Thereafter *IgMsec* expression decreased slightly but was maintained at elevated levels, until the end of the experiment. Although these data suggest an involvement of humoral responses in the infection with *S. molnari*, it remains to be determined whether the produced antibodies are specific or if the infection merely induces a polyclonal IgM hypersecretion with no effective binding of the parasite. Polyclonal activation is a strategy used by parasites to escape the host-specific immune response by means of diluting pathogen-specific antibodies while increasing irrelevant antibodies (e.g. [43–47]). To elucidate this question, BS of *S. molnari* were used as antigen in western blotting with sera of experimentally infected fish. The parasite lysate yielded 6 apparent protein bands in sizes of 13, 16, 28, 48, 55 and 70 kDa (Fig. 3). However, after membrane transfer, we were able to demonstrate specific IgM binding to a parasite protein of 16 kDa, from 28 dpi onwards, with increasing intensity over time, peaking at 63 dpi (Fig. 3, blot and red line in figure). No signal was detected in the serum of fish injected with PBS. Taken together, these data provide strong evidence for the induction of a specific carp IgM antibody response to *S. molnari*.

### Cytokine expression in response to *S. molnari* shows strong involvement of il-10

**Fig. 4:**
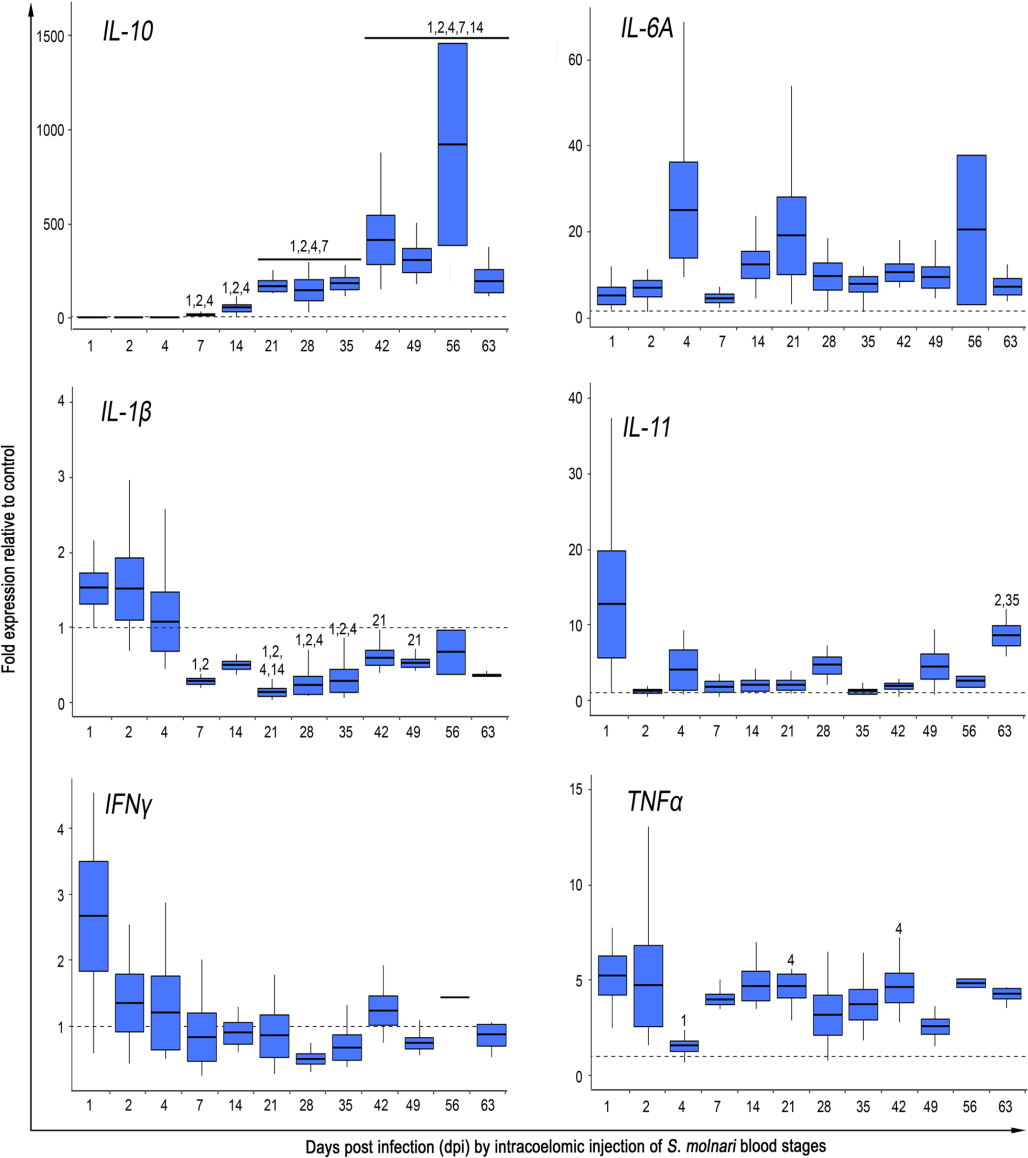
Cytokine expression profile as a response of common carp to *S. molnari* intraperitoneal blood stage injection showing fold change expression relative to the control group over time. Note the massive increase of *il-10* expression from 7 dpi onwards with maximum values reached at 56 dpi. This coincides with *il-1β* and *ifnγ* inhibition. Statistical analyses are based on data-specific LMM developed in R with significance differences shown relative to other sampling days (numbers above box plot; significance levels detailed in Additional file 1: Figure S3). Box plots show mean ± standard deviation (box) with maximum and minimum ranges (lines)

The expression of six pro- and anti-inflammatory cytokines was studied in the head kidney of experimental and control fish (Fig. 4). Within the first week after the injection of *S. molnari* into the peritoneal cavity (latent period of infection), we observed only a mild increase in the expression of the pro-inflammatory cytokines driving the acute phase of the immune response. After this initial stage, expression followed these patterns: (i) *il-1β*, *ifnγ* and *il-11* expression was similar or lower than in the control; (ii) *il-6* and *tnfα* expression was elevated throughout the experiment; and (iii) expression of *il-10* increased gradually to the highest levels at day 56. More specifically, the expression of *ifnγ*, which orchestrates innate and adaptive responses against intracellular pathogens and supports the development of pro-inflammatory macrophages [48], was highest at 1 dpi with 2.8-fold upregulation, but decreased to levels below those observed in control fish as early as 7 dpi. Similarly, the expression of *il-1β*, a key regulator of inflammation, increased only slightly during first three days with a maximum 1.5-fold increase at 2 dpi and a subsequent decrease below the expression level observed in control fish, likely indicating inhibition, from 7 dpi onward and for the remainder of the experimental infection (lower expression over time when compared to initial dpi, LM: *F*_(11,44)_ = 9.1, *P* < 0.001 to 0.014). The expression of *il-11* has previously been shown to be involved in the induction of acute phase proteins and in the maturation of thrombocytes [49]. This member of the *il-6* family reached its highest (12-fold) expression at 1 dpi but decreased to control levels by 2 dpi and remained only slightly elevated, with an increase on day 28, 49 and day 63. Conversely, another mediator of acute phase response, *il-6* exhibited increased expression from 1 dpi and throughout the whole study, with peaks occurring at 4 dpi (25-fold expression), 21 dpi (19-fold expression) and 56 dpi (21-fold expression). The infection also induced a 4- to 5-fold increase in the expression of *tnfα* with occasional decreases at 4 and 49 dpi, indicating a prolonged activation of innate arm of the immune response [50]. In contrast to the above mentioned cytokines, the expression of the anti-inflammatory *il-10* remained unchanged during the first week, but increased gradually with a 3-fold upregulation at 7 dpi, a 17-fold upregulation at 14 dpi and a subsequent increase over time, reaching up to 1456-fold expression in individual fish (average 921-fold, 56 dpi; Fig. 4; LM: *F*_(11,43)_ = 39.4, *P* < 0.001 to 0.047).

## Discussion

For myxozoans, knowledge of the presporogonic intrapiscine development is scarce and detailed information on the course and location of all stages is limited to a few host–parasite systems. The unavailability of such data is explained by problems related to running experimental studies, i.e. lack of known myxozoan life-cycles, limits regarding the production of infective stages in invertebrate hosts, or lacking knowledge of the seasonality of highly infective spore concentrations in natural habitats. In this study, we bypassed these problems as well as potential co-infections by producing *S. molnari* BS in an *in vivo* infection system in laboratory-reared specific pathogen-free (SPF) carp.

Using qPCR, *S. molnari* was first detected in the blood at 14 and 21 dpi, indicating a prolonged period of covert existence in the host. In natural infections of other myxozoans, invasion is fast and initial cycles of parasite multiplication close to the invasion site are initiated immediately [22, 25, 51, 52]. It may be argued that, due to the artificial intraperitoneal introduction route, *S. molnari* requires additional time to find its entrance to the vascular system. However, we demonstrated that the majority of CSFE-labeled parasites had departed the peritoneal cavity within 40 hpi, possibly *via* the peritoneal efflux [53], or surface receptors, which allow it to actively navigate through host tissues to a desired location [54]. We suggest that *S. molnari* enters specific host cells and starts intracellular proliferation, prior to extracellular proliferation in the blood. Our hypothesis is partially supported by a 2-week delay in the detection of first leukocyte responses, yet the increased expression of pro-inflammatory cytokines in the head kidney during the first four days of infection suggest an activation of systemic immune response on first contact, albeit a mild one. Despite our efforts, the exact location of the parasite before its occurrence in the blood at 14 to 21 dpi remains enigmatic, as parasite DNA was not found in any of the tested tissues.

Once in the blood, cell doublets, the main presporogonic proliferation stage in myxozoans [55] are predominant. Parasite appearance in the blood coincides with the increase in the number of neutrophils and monocytes, which are essential for the host responses *via* production of reactive oxygen species, netosis, phagocytosis, activation of the immune system and antigen presentation [56–58]. Such changes in the mobilization of leukocytes were described previously in infection experiments with other myxozoan parasites, although a direct comparison of these findings is rather complicated, as the parasites differ in their organ tropism, the timing of the infection and chosen sampling points. In gilthead sea bream, a mobilization of mast cells and Ig^+^ cells to the intestinal infection site, together with a decrease in the number of granulocytes in the head kidney was observed 40 days after exposure to *Enteromyxum leei*, the agent of myxozoan emaciation disease [59]. Similarly, infection with *Kudoa thrysites* in the muscle of Atlantic salmon leads to the activation of macrophages and dendritic cells, i.e. phagocytes and antigen presenting cells [33]. In our study, the elevated expression of cytokines and recruitment of monocytes and neutrophils to blood indicates successful recognition of the parasite and activation of inflammatory responses, yet, the killing capacity of these cells proved insufficient. Partially, this could be attributed to motility, which *S. molnari* uses to avoid cellular contact with monocytes and neutrophils [17] but also to additional strategies used by other myxozoan parasites [60].

We identified 35 dpi as a key point in the course of the *S. molnari* infection. At this time, the number of parasites was reduced in all tissues examined, with an exception of the gills. Most likely, this is mediated by the presence of *S. molnari*-specific antibodies, first detectable in the serum of infected fish at 28 dpi. The observed reduction in parasite numbers can likely be attributed to opsonization of the parasite with the specific antibodies, activation of complement and cell-mediated killing by leukocytes [61]. Interestingly, these effector functions did not clear the parasites in the gill, which might be explained by the partially intracellular nature of *S. molnari* spore-forming stages in the gills, with host epithelial cells serving as a protective niche, where the parasite remains unharmed [13, 62, 63].

Despite the presence of specific antibodies in the serum, parasite numbers recovered during the following days of infection and *S. molnari* invaded the liver, where the largest parasite quantities were observed. Liver stages are larger, and we demonstrated that blood stages with more than nine cells only occur once the liver becomes infected and likely due to release of these large stages from this host reservoir. After re-entry into the blood, it is unclear if further parasite proliferation occurs or if LS just use the blood stream for transport to the gills. However, at this stage, a second peak of BS occurrence was observed at 42 dpi, which is lower than the previous one but mirrored in all organs due to blood circulation within them, the same as at 28 dpi. Complex migration patterns with portals of entry different from the target tissue and several distinct developmental stages in the fish host are common to the Myxozoa, but proliferation in the blood is a characteristic specific only to sphaerosporids [64–67]. In *S. dykovae*, further development of BS occurs in the swim bladder, with secondary cells containing two tertiary cells, a stage identical with the earliest stages of sporogony in the kidney tubules [55, 64], and these triplets were also detected in *Sphaerospora truttae* [68]. In *S. molnari*, the typical cell triplets were not observed in BS or LS, and it is assumed that they will only appear later, during sporogonic development in the gills. This suggests that the LS is a larger but not different stage of parasite development. It is, however, likely that nutrients are being taken up from the liver, as the primary cell membrane is almost indistinguishable (Fig. 1), indicating close contact with host cells and potentially active feeding mechanisms. Myxozoan plasmodia have been shown to exhibit a distinct periphery with high pinocytotic activity [69–71]. The survival and growth of *S. molnari* in the liver implies an effective use of exploitation and disguise mechanisms, similar to *Plasmodium* multiplication in the liver which is clinically silent because the parasite synthesizes host bulk phospholipids [72] and hijacks specific host pathways to promote its own development [73], while a number of mechanisms prevent host immune response and phagocytosis [74].

Between 42 and 49 dpi, the number of parasites decreased in all studied organs and remained low for the rest of the experiment, marking the onset of a chronic infection. However, despite the reduced number of parasites, we still observed increased numbers of thrombocytes and monocytes, possibly involved in parasite clearance by phagocytosis [75]. Alternatively, the increased number of thrombocytes might be related to the resolution of inflammation and wound healing, as activated platelets can be potent drivers of anti-inflammatory responses [76].

The changes in leukocyte composition were complemented by changes in the expression of selected cytokines with known function in the acute phase of the immune response. Peritoneal injection of the parasite induced an immediate increase in the expression level of some cytokines, suggesting the recognition of *S. molnari* by pattern recognition receptors, such as toll-like receptors (TLRs). Although the interaction of TLRs with myxozoans has not been studied, their importance in the recognition of fish parasites has previously been demonstrated [27, 77]. Notably, the expression of *il-1β* and *ifnγ* increased only to a limited extent early after the injection of *S. molnari* and thereafter was inhibited (*il-1β*) or close to the levels observed in control fish (*ifnγ*). Similarly, inhibition of *il-1β* has been previously reported in the kidney of brown trout infected with *Tetracapsuloides bryosalmonae*, the agent of proliferative kidney disease (PKD) [78], in the intestine infected with *E. leei* [79] or *C. shasta*, the agent of enteronecrosis or gut-rot disease in salmonids [32]. Low expression of *ifnγ* was previously reported in the kidney during the early stages of PKD (slightly increased by 50 dpi; [80]), while other myxozoan models suggest its increase and possible contribution to the cytotoxic activities [81, 82]. The timing of the downregulation of these genes is in accordance with the increasing expression of *il-10* [83]. Additionally, the transition from low pro-inflammatory to strong anti-inflammatory reaction has been described not only in myxozoans [29, 32, 84] but also in other parasitic infections [85]. These findings suggest that despite initial activation of the pro-inflammatory response, the extent of the inflammation is limited shortly after.

Meanwhile, *il-6* and *tnfα* were upregulated throughout the study indicating a prolonged activation of innate immunity. The increase of *tnfα* has been reported in other models including carp infected with *Trypanosoma borreli* [86] and in all examined organs of fish infected by *E. leei* [82]. Notably, *tnfα* does not only play the classical role by stimulating the production of a number of genes associated with inflammation, enhancing the phagocytic activity of leukocytes, regulating homing, proliferation and migration [50], but it also exhibits lectin-like activity towards parasites such as *Trypanosoma brucei* [86]. However, the effect of *tnfα* is not always beneficial, as the excessive production of *tnfα* was shown to be associated with the development of lesions and loss of barrier function during enteromyxosis in turbot [87]. *il-6* is known for its pleitropic effect playing a pivotal role during the transition from innate to acquired immunity. In fish, it has been reported to promote macrophage growth and production of antimicrobial proteins [84], while at the same time supporting antibody production [88, 89]. Possibly, in the *S. molnari* infection it might be related to the attraction of monocytes, secretion of IgM from 21 dpi onward and the resolution of the inflammation in the final stages. Involvement of *il-6* has also been described in *E. leei* infected gilthead sea bream [82] and *C. shasta* infected Chinook salmon [32].

From the selected cytokines, the anti-inflammatory *il-10* underwent the strongest upregulation, reaching up to 1456-fold increase in expression compared to control fish, at 56 dpi. As carp has two paralogues of *il-10* which have almost identical gene structure, synteny, protein sequence and exert identical biological activities *in vitro*, it is worth noting that the primers used in the present study were targeting the *il-10b* paralogue, which is preferentially upregulated upon infection [83]. Traditionally, *il-10* is not only associated with the control of excessive immune responses but in cell cultures obtained from *T. borreli*-infected carp, *il-10* promotes the proliferation, differentiation and antibody secretion by the IgM^+^ B cells [90]. Importantly, a growing evidence from other disease models suggest that the protective function of *il-10* can be exploited by pathogens [91]. In infection with other Myxozoa, high levels of *il-10* were induced by *C. shasta* [32], *T. bryosalmonae* [78] and also in the intestine, but not in head kidney of gilthead sea bream infected *E. leei* [82]. These results point to *il-10* functioning as a surrogate of myxozoan infections and question its role in host-pathogen interaction. Possibly, the expression of *il-10* can be explained by two scenarios. In the traditional understanding, *il-10* is induced by the immune system itself in an effort to minimize the collateral damage caused by neutrophils and monocytes fighting the growing number of *S. molnari*. Alternatively, *S. molnari* uses an unknown mechanism to induce *il-10* as an immunomodulation strategy to deactivate the effector capacities of the immune system. The present data does not exclude any of these scenarios and future studies will investigate whether high concentrations of *il-10* are a cause or a consequence of high pathogen burdens.

Using this model of *S. molnari* infection, we investigated the kinetics of antibody responses in infected fish, including their expression levels, level of specific antibody and number of B lymphocytes, over time. As the present model bypasses the natural route of infection *via* mucosal surfaces, where the activation of mucosal immunoglobulins could be expected, we focused solely on the response of systemic IgM [46, 92, 93]. Previously, myxozoan infections were reported to show a delayed development of antibody responses, with specific immunoglobulins detectable after 6–8 weeks [30]. However, we observed an early onset of the reaction to *S. molnari*, with the first significant increase in IgM levels detectable at 14 dpi. Such timing is in agreement with the known time span for antibody responses in common carp towards model antigens such as LPS or KLH, which at 21 °C takes 10–28 days [94]. Expression data were further supported by a 9-fold increase in the absolute numbers of IgM^+^ B cells in the blood. Such a prominent reaction of B lymphocytes with excessive expression of IgM points to a possible hypergammaglobulinemia, resulting in a polyclonal expansion and production of immunoglobulins with only a minor proportion of specific antibodies, a phenomena that has been reported in two other myxozoan infections [46, 95–97], in other parasites of fish (*T. borreli* [98]) and mammals (*Trypanosoma cruzi* [44]; *Plasmodium chabaudi* [45]).

To elucidate the nature of the B cell responses, we investigated the presence of specific antibodies to *S. molnari* by SDS PAGE of parasite antigens and subsequent western blotting with sera of infected fish, which identified a single protein of 16 kDa. While the identity of this protein remains to be determined, it is noteworthy that an antigenic protein of the same size (and an additional 165 kDa protein) was characterized in *E. leei*, using a similar approach [99]. The two proteins could well correspond to common antigens shared among myxozoans. Estensoro et al. [97] showed the *E. leei* antigen to be a glycoprotein, and suggested a minicollagen; however, minicollagens would unlikely be expressed in proliferative stages of *S. molnari* as they are essential components of polar capsules and cnidarian stinging cells [100, 101], expressed during spore formation. Nevertheless, while the western blot clearly demonstrated the acquisition of specific immunity of carp against *S. molnari*, the low titer of specific antibodies in combination with an extremely high numbers of circulating B cells (up to 9-fold increase in number) indirectly confirms the suspicion that *S. molnari*, like other myxozoan parasites, manipulates B cells responses.

## Conclusions

Our study demonstrates an activation of innate and adaptive humoral immune responses of common carp to the myxozoan *S. molnari*, during early stage of infection and parasite proliferation. We show that myxozoans use important immunomodulatory and immune evasion strategies such as intracellular disguise, motility, polyclonal activation of B cell responses and skewing of host response to an anti-inflammatory phenotype, in order to be able to successfully proliferate in their fish hosts and produce infective spore stages to continue their life-cycle. We believe that the present comprehensive characterization of host and parasite interactions between *S. molnari* and common carp represents a solid base for further research investigating newly raised questions in our *in vivo* model, with a focus on specific immune responses and universal myxozoan antigenic proteins that could in the future be used for targeted therapies and potential vaccine design.

## Methods

### Experimental infection of fish and sampling procedure

In May 2015, fertilized eggs of common carp (*Cyprinus carpio*) were obtained from the Faculty of Fisheries and Protection of Waters of the University of South Bohemia. They originated from a carp population reared in production ponds in Vodňany (Czech Republic). Eggs were stocked and fish were reared in an experimental recirculation system (UV filtration, ozone) at the animal facility of the Institute of Parasitology, Biology Centre of the Czech Academy of Sciences. Water temperature was kept at 21 ± 1 °C and fish were fed a commercial carp diet (Skretting, Stavanger, Norway) at a daily rate of 2% of biomass. In April 2016, 57 fish (weight 15 ± 4 g), were intraperitoneally injected with motile blood stages of *S. molnari*, isolated from whole blood of an infected carp population aged 0+. Isolation of the parasites was performed by centrifugation of whole blood from 18 donor fish (0+) in heparinized hematocrit capillaries (4000×*g*, 5 min). A mixture of white blood cells and parasite stages was collected after breaking of the capillary tubes. The cells were washed in PBS and after centrifugation (800×*g*, 5 min) they were resuspended, quantified and adjusted to 370 parasites/µl. A volume equaling 14,800 parasites per fish (averaging 1000 parasites per g of body weight, 40 µl of parasite/host cell solution) was intraperitoneally injected into SPF receptor fish. Thirty-six control fish were injected with 40 µl of PBS. This experiment was repeated with 10 specific-pathogen-free carp, to obtain information on B cell numbers by cytometry on a weekly basis. All fish were anesthetized with clove oil before bleeding or injection. Experimental and control carp were maintained in 3 isolated aquaria of 80 liters (2× infected, 1× control), with aeration, filtration, and weekly water changes. Sampling of fish was performed at 1, 2, 4 and 7 days post-infection (dpi) and thereafter weekly until week 9 post-infection, with 5 experimental and 3 control fish sampled per date. No mortalities occurred during the experiment. On each sampling date, blood was drawn with a heparinized syringe and full hematological analyses as well as flow cytometry were performed. Two blood smears were produced and fixed with methanol. Samples of blood, gills, kidney, liver and muscle were stored in TNES urea [102] for DNA extraction; the head kidney was fixed in RNAlater (Ambion, Europe Ltd, Huntingdon, UK) and frozen. Sera were collected and frozen after spinning the remainder of the blood sample at 4000×*g* for 10 min.

### Hematology

Full blood was diluted in Hayem’s solution (1:200) and blood cells were counted in a Bürker chamber, with the methodology detailed in Additional file 1: Figure S3. Two replicate blood smears per fish, fixed with methanol, were stained with Kwik–Diff (Richard Allen Scientific, San Diego, USA), according to the manufacturer’s recommendations and permanently mounted with Eukitt (Merck, Darmstadt, Germany). Two times 100 differentially stained cells (leukocytes, thrombocytes, *S. molnari* blood stages but excluding erythrocytes) were identified morphologically, based on known host and parasite features [13, 17, 103]. The compacted erythrocyte cell volume relative to whole blood volume (hematocrit) was measured in 2 capillaries per fish, drawn from whole blood and centrifuged at 10,000×*g* for 5 min. Hemoglobin content was estimated by spectrophotometry (540 nm) after lysis of cells and conversion of hemoglobin to cyanmethemoglobin, using Drabkin’s solution (Sigma; 1:250 dilution of blood:Drabkin’s; duplicate measurements). Hemoglobin concentrations were calculated from absorbance levels according to y (g l^−1^) = (A_540_–A_BLANK_) W_Hb_ *F*_*D*_ (C_E_ *d* 1000)^−1^, where A_540_ is the absorbance at 540 nm, A_BLANK_ is the absorbance of pure Drabkin’s reagent at 540 nm, W_Hb_ is the molecular mass (human hemoglobin tetramer; = 64,458), *F*_*D*_ is the dilution factor (= 250), C_E_ is the millimolar extinction coefficient of tetrameric cyanomethhemoglobin at 540 nm (= 44), *d* is the vial light path in cm (= 1) and 1000 converts from mg to g [104].

### Flow cytometry

To gain insights into the number and proportion of B lymphocytes from 7 dpi onwards and throughout the infection we adapted a protocol for the flow cytometric analysis of the full blood described previously [105]. Briefly, 2 µl of blood of control and infected fish were washed with cold RPMI and stained for 20 min with a monoclonal antibody recognizing the heavy chain of carp IgM (1µg/ml) (Aquatic Diagnostics Ltd, Stirling, UK), followed by staining with goat-anti-mouse IgG Alexa Fluor 488 (2 µg/ml; Thermo Fisher Scientific, Pardubice, Czech Republic). The samples were washed twice and resuspended in 200 µl of RPMI. The proportion and total number of IgM^+^ B cells were determined using BD FACSCanto II (BD Biosciences, Prague, Czech Republic). Each sample was acquired for 30 s with a flow rate of 60 µl/min.

### Parasite localization and quantification

*Sphaerospora molnari* was quantified in blood, gills, kidney, liver and muscle tissues, using real-time quantitative PCR which was performed using a LightCycler® 480 (Roche, Prague, Czech Republic). DNA was extracted using a modified phenol/chloroform extraction protocol [106], including RNAse A treatment at 50 µg/ml, for 30 min at 37 °C, before ethanol precipitation. DNA concentration and purity was estimated using a NanoDrop spectrophotometer (Thermo Fisher Scientific) and dilutions of 100 ng/µl were prepared using RNAse/DNAse-free water. *Sphaerospora molnari* stages were quantified using a TaqMan-based qPCR assay base on the *SSU* rDNA sequence of the parasite (GenBank accession number JX431511). Briefly, each 25 µl reaction contained 100 ng of DNA, 0.4 µM forward and reverse primers, 0.2 µM 5′ FAM and 3’BHQ1-labelled Taqman probe and 12.5 µl of LightCycler® 480 Probes Master (Roche) as detection chemistry. Samples were run as duplicates and Ct values for each sample were recalculated based on the average reaction efficiencies for each primer set, which were obtained upon comparative quantitation analysis. Ct values were compared after adjustment using an inter-plate control sample. Parasite *SSU* rDNA copy numbers were calculated relative to the sample with the highest parasite concentration (set to 100) and normalized relative to host *β*-actin (housekeeping gene). All qPCR primers and probes are detailed in Additional file 1: Table S1. Multicellular blood stages were furthermore quantified on Kwik–Diff-stained blood smears (see above) to be able to relate qPCR values to parasite numbers of varying cell composition in the blood.

In order to elucidate the early parasite migration/uptake from the intraperitoneal injection site, in a second trial we labelled isolated *S. molnari* blood stages (BS) with carboxyfluorescein succinimidyl ester (CellTrace™ CFSE Cell Proliferation Kit; Thermo Fisher Scientific) at 1:10,000 concentration, washed the cells twice with RPMI medium and injected 100,000 BS per fish into the peritoneal cavity of 15 SPF carp. Peritoneal lavages were performed at 16 h, 40 h, 7 days and 21 days post-injection. Percoll-isolated leukocyte + parasite preparations (57%/34% Percoll interphase collected after centrifugation at 400×*g*, 20 min) of head kidney, spleen and blood were also prepared and the recovered cells were analyzed by flow cytometry (number of CSFE-labelled *S. molnari* stages per 100,000 cells). The cells isolated from blood were microscopically screened for motile BS at 21 dpi. Three fish were sampled at each time point.

### IgM expression in head kidney

Transcriptomic data obtained from a mixture of white blood cells and *S. molnari* blood stages was mined for IgM heavy chain sequences of *C. carpio*. Two isoforms were found, one with a transmembrane domain (on B cell/plasma cell surface; GenBank: MH352353) and one with a secretory tail (secreted form; GenBank: MH352354). We developed TaqMan qPCR assays to differentiate between the expression of the secretory tetramer (IgMsec) and the membrane-bound monomer (IgMmem) (Additional file 1: Table S1). IgM expression was determined in the head kidney of experimental and control fish, after extraction of total RNA from the RNAlater-fixed samples using the NucleoSpin RNA (Macherey-Nagel, Düren, Germany) and cDNA preparation with the Transcriptor High Fidelity cDNA Synthesis Kit (Roche Diagnostics). Both RNA as well as cDNA quality and quantity was determined by NanoDrop measurement. qPCRs were performed on samples adjusted to 100 ng/µl cDNA concentrations and relative to carp *β*-*actin* (Additional file 1: Table S1), using the same quality and compatibility measures as above.

### Detection of *S. molnari* specific antibodies by western blot

In order to determine specific antibody responses induced by the infection with *S. molnari*, we performed western blotting with sera of infected carp. For antigen preparation, blood stages of *S. molnari* were obtained from infected fish at the peak of infection (28 dpi, see “Results”). Whole blood was diluted in 5 ml of cold RPMI and layered onto Ficoll. Following gradient centrifugation at 800×*g* for 20 min, a mixture of white blood cells and parasites was collected on the top of the Ficoll layer and washed once with cold RPMI. Parasites were enriched by allowing adhesion of white blood cells to a cell culture dish for 30 min, before being pelleted by centrifugation and resuspended in cold RPMI. The purity of the resulting parasite mixture was assessed under the microscope. The parasite preparation was mixed with 2× Laemmli buffer (Bio-Rad, Prague, Czech Republic) and used as an antigen for the western blotting following the manufacturer’s protocol. Proteins divided by SDS PAGE in a 12% gel were transferred on PVDF membrane using the Trans-Blot Turbo™ Transfer System. Following blocking with 8% of dry milk dissolved in PBS-Tween, the membrane was cut into strips, which were exposed to the pooled sera of infected fish (5 fish per time point) diluted 1:100 in PBS-Tween. After washing with PBS-Tween, mouse anti-carp IgM antibody (see above) was used as a primary antibody to visualize specific carp IgM bound to parasite protein, followed by the incubation with secondary HRP-labeled goat anti-mouse IgG (Thermo Fisher Scientific). Finally, after the addition of ELC substrate, the western blot was visualized on a ChemiDoc Imaging System (Bio-Rad).

### Cytokine gene expression

To obtain further insights into the regulation of immune responses the expression of a panel of pro- and anti-inflammatory cytokines was analyzed by qPCR (Additional file 1: Table S1). The head kidney was selected for the analysis as it is considered the principal immune organ in fish combining primary and secondary immune function [107]. Furthermore, the slow blood flow through its stroma presumably allows a direct contact with the parasite once it appears in the blood. The reaction mix contained 0.26 µM forward and reverse primers, 100 ng of cDNA and 7 µl of FastStart Universal SYBR Green Master Mix (Roche) in 16 µl reactions. All samples were run in duplicates, applying the same quality standards as above. The relative expression ratio of each sample was calculated according to Pfaffl [108], based on the take-off deviation of sample *versus* controls at each time point and normalized relative to host *β*-actin (housekeeping gene).

### Statistics

Cytokine and IgM expression differences were evaluated with linear models (LM), using log-transformed response variables. Thereafter, multiple pairwise comparisons evaluating the influence of fixed effect (dpi) were obtained using Tukey’s all-pair comparisons, applying the Bonferroni correction to adjust the *P*-values (package *multcomp*, v.1.3-3; [109]). To investigate differences in cells numbers (lymphocytes, B cells, monocytes, neutrophils and thrombocytes) between days and groups, the data were analyzed using linear models with log-transformed response variables and an interaction of the fixed factors dpi and group (control/experimental). Thereafter, pairwise comparisons evaluating the influence of the fixed effects on cell numbers were obtained as explained above. Parasite numbers were compared between sampling dates using the Kruskal Wallis H test. All analyses were conducted in R (R Core Team, v.3.4.2.). Graphs were prepared in R and colored/assembled in Adobe Photoshop.

## Additional file

**Additional file 1: Figure S1.** Full parasite data obtained by qPCR. **Figure S2.** Hemoglobin and hematocrit changes in blood parameters over time during *S. molnari* infection. **Figure S3.** Blood cell counts in Bürker chamber. **Table S1.** qPCR primers and probes developed for or used in the present study.

## Abbreviations

BS: blood stage (of *S. molnari*)
Ct value: cycle threshold value
dpi: days post infection
hpi: hours post infection
IgM: immunoglobulin M
LMM: linear mixed model
LM: linear model
LS: liver stage (of *S. molnari*)
Mya: million years ago
SDS PAGE: sodium dodecyl sulfate-polyacrylamide gel electrophoresis
PBS: phosphate-buffered saline
PVDF: polyvinylidine fluoride
