# Supplementary material for: The kinetics of cellular and humoral immune responses of common carp to presporogonic development of the myxozoan Sphaerospora molnari
Source: Parasit Vectors. 2019 May 6;12:208. doi: 10.1186/s13071-019-3462-3 (PMC6501462; doi:10.1186/s13071-019-3462-3)
Supplement: Supplementary file 1 — Additional file 1: Figure S1. Full parasite data obtained by qPCR. Figure S2. Hemoglobin and hematocrit changes in blood parameters over time during S. molnari infection. Figure S3. Blood cell counts in Bürker chamber. Table S1. qPCR primers and probes developed for or used in the present study. [file 13071_2019_3462_MOESM1_ESM.docx]

**Additional file 1**

**Figure S1:** Full parasite data obtained by qPCR


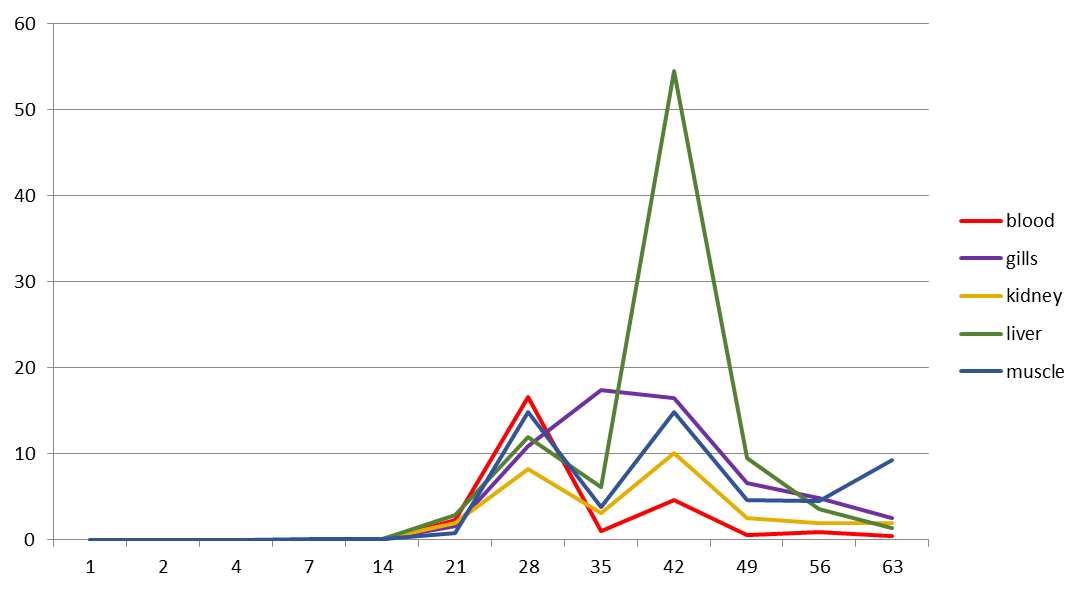

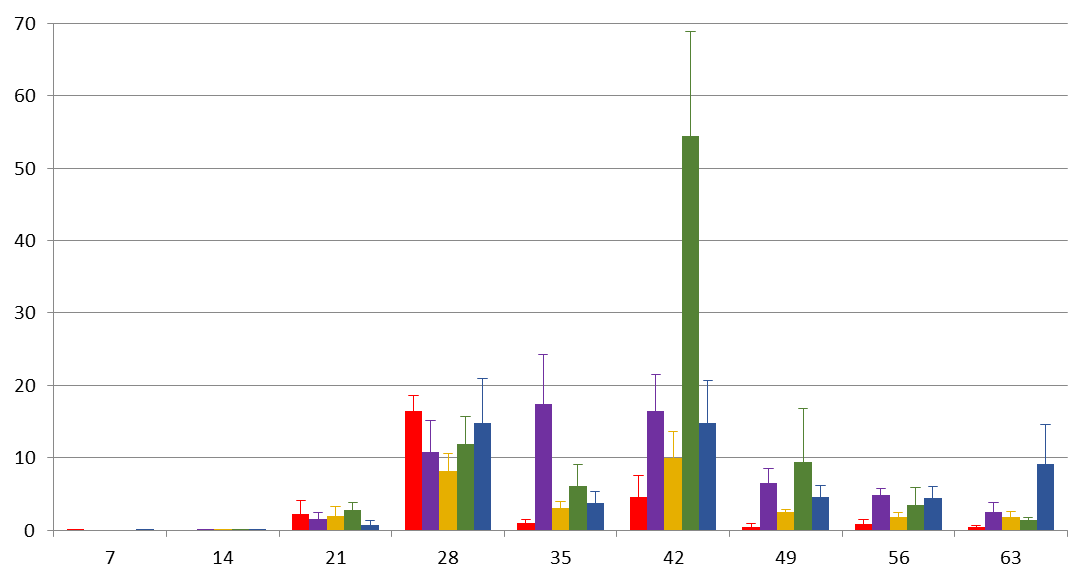


Relative quantities of parasites (highest sample set to 100)

dpi

Quantification of *S. molnari* by qPCR in different host organs and on different days post intracoelomic injection (dpi) into common carp. Each value is an average of 5 fishes, error bars represent standard error. Quantification is relative to the lowest Ct value obtained (a liver sample on day 42), which was set to 100.

**Figure S2:** Hemoglobin and hematocrit changes in blood parameters over time during *S. molnari* infection


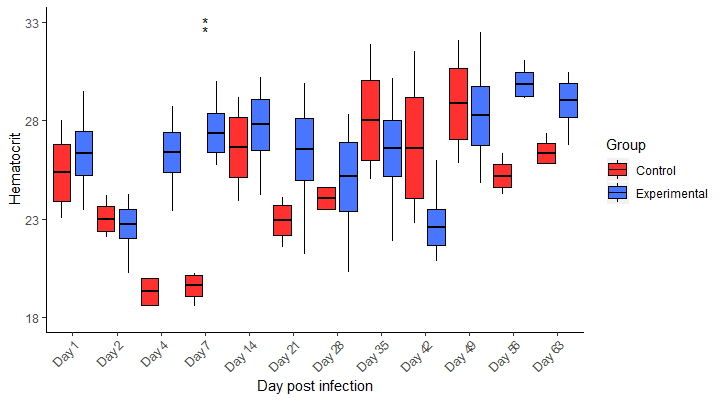

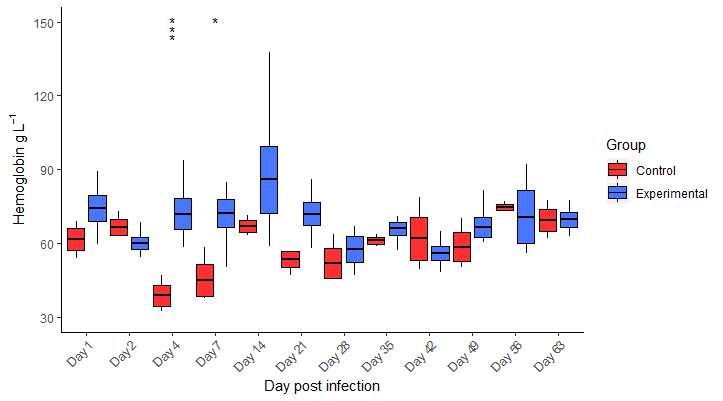


Hematocrit and hemoglobin values did not decrease significantly in experimental fish injected with *S. molnari* when compared with the control group. However, there was a significant decrease in these parameters 4 and 7 dpi in the control group. This change is unexplained.

**Figure S3:** Blood cell counts in Bürker chamber


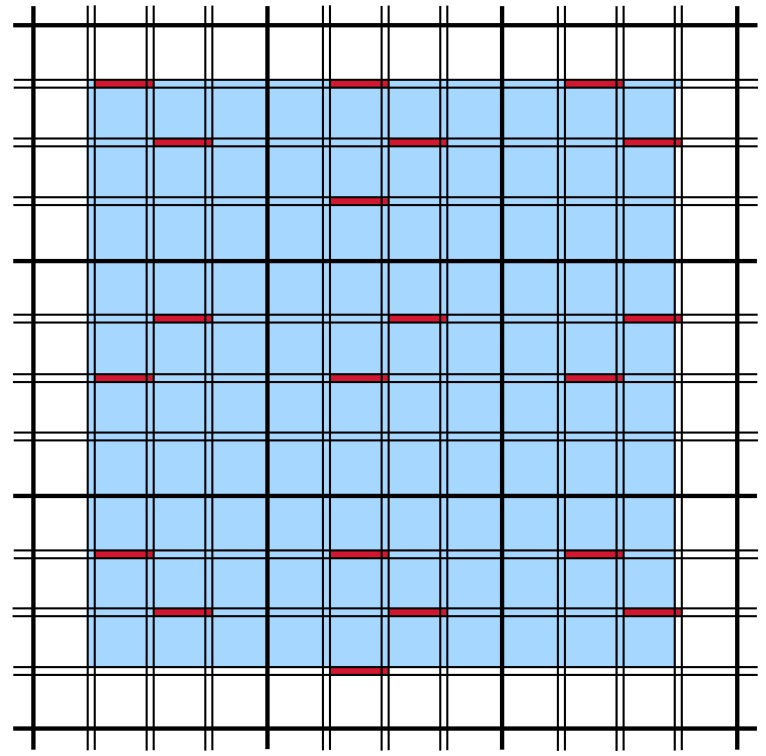


Full blood was diluted in Hayem’s solution (1:200) and erythrocytes were counted in small fields (marked in red), while leukocytes and thrombocytes were counted in a large square (marked in light blue). All counts were performed 2-3 times for each blood sample and averaged thereafter. Thrombocytes and leukocytes can be difficult to differentiate when unstained as they are similar in size. In order to avoid misidentification by different researchers performing the counts (n=2-3 at each sampling event) thrombocytes were included into the total leukocyte count and deducted after determination of thrombocyte numbers in the differential blood cell counts on stained slides. The leukocyte+thrombocyte count was divided by 2 to obtain the number of these cells *10^3^ µl^-1^ of blood, the erythrocyte number represents the number of erythrocytes *10^4^ µl^-1^.

**Table S1:** qPCR primers and probes developed for or used in the present study

| **Target gene** | **Assay** | **Primer (F/R)/probe (P)** | **Sequence (3’ to 5’)** | **Amplicon size** | **Reference** |
| --- | --- | --- | --- | --- | --- |
| *S. molnari* SSU rDNA | TaqMan | SmolSSU_qpcrF | TGCTGTGTGACGTGTGTCC | 106bp | JX431511; this study |
|  |  | SmolSSU_qpcrR | ATGCATGTGTGAGCGTGATT |  |  |
|  |  | SmolSSU_qpcrP | BHQ1-GCCTGTGCTCAATGTAGCAC-FAM |  |  |
| IgMsec | TaqMan | IgM-Cc_secF | TCGTATTAGCACCCCCAGAG | 106bp | MH352354; this study |
|  |  | IgM_Cc_secR | TCATCAGCAAGCCAAGACACA |  |  |
|  |  | IgM_Cc_sec_P | BHQ1-ACAAAAAGGGTGAACCGATG-FAM |  |  |
| IgMmem | TaqMan | IgM_Cc_memF | GCTAGAGCATCCCTGTACG | 94bp | MH352353; this study |
|  |  | IgM_Cc_memR | CAAGGTGATGAGGAACAGGAA |  |  |
|  |  | IgM_Cc_memP | BHQ1-TTGCAAACACTGCCATAACC-FAM |  |  |
| *β*-actin | TaqMan | Actin_Cc_DNA_F | AGGTATGGAGTCTTGCGGTA | 80bp | M24113; this study |
|  |  | Actin_Cc_DNA_R | ACAGGTCCTTACGGATGTCG |  |  |
|  |  | Actin_Cc_DNA_P | BHQ1-TGAGACCACCTTCAACTCCAT-FAM |  |  |
| IL-1*β* | SYBR Green | IL-1*β*-F | AAGGAGGCCAGTGGCTCTGT | 69bp | AJ245635; [1] |
|  |  | IL-1*β*-R | CCTGAAGAAGAGGAGGCTGTCA |  |  |
| IL-6A | SYBR Green | IL-6A-F | CAGATAGCGGACGGAGGGGC | 191bp | KC858890; [1] |
|  |  | IL-6A-R | GCGGGTCTCTTCGTGTCTT |  |  |
| IL-10 | SYBR Green | IL-10-F | CGCCAGCATAAAGAACTCGT | 103bp | AB110780; [2] |
|  |  | IL-10-R | TGCCAAATACTGCTCGATGT |  |  |
| IL-11 | SYBR Green | IL-11-F | CAGCAGCACAGCTCAGTACCA | 96bp | AJ632159; [3] |
|  |  | IL-11R | AGCCTCTGCTCGGGTCATCT |  |  |
| TNFα | SYBR Green | TNFα-F | GCTGTCTGCTTCACGCTCAA | 106bp | AJ311800; [4] |
|  |  | TNFα-R | CCTTGGAAGTGACATTTGCTTTT |  |  |
| INFγ 2A/2B | SYBR Green | INFγ-F | CGATCAAGGAAGATGACCCAGTC | 73bp | AM168523; [4] |
|  |  | INFγ-R | GTTGCTTCTCTGTAGACACGCTTC |  |  |
| *β*-actin | SYBR Green | *β*-actin-F | GCTATGTGGCTCTTGACTTCGA | 89bp | M24113; [3] |
|  |  | *β*-actin-R | CCGTCAGGCAGCTCATAGCT |  |  |

**References**

1. Piazzon MC, Savelkoul HS, Pietretti F, Wiegertjes GF, Forlenza M. Carp Il10 has anti-inflammatory activities on phagocytes, promotes proliferation of memory T cells, and reuglates B cell differentiation and antibody secretion. J Immunol. 2015;194:187-99.

2. Piazzon MC, Wentzel AS, Wiegertjes GF, Forlenza M. Carp IL10a and IL10b exert identical biological activities in vitro, bur are differentially regulated in vivo. Dev Comop Immunol. 2017;67:350-60.

3. Joerink M, Ribeiro CM, Stet RJ, Hermsen T, Savelkoul HF, Wiegertjes GF. Head kidney-derived macrophages of common carp (*Cyprinus carpio* L.) show plasticity and functional polarization upon differential stimulation. J Immunol. 2006;177:61-9.

4. Embregts EWE, Rigaudeau D, Veselý T, Pokorová D, Lorenzen N, Petiti J, Houel A, Dauber M, Schütze H, Boudinot p, Wiegertjes FG, Forlenza M. Intramuscular DNA vaccination of juvenile carp against spring viremia of carp virus induces full protection and establishes a virus-specific B and T cell response. Front Immunol. 2017;8:1340.
